# Cross Sectional Survey of Antenatal Educators’ Views About Current Antenatal Education Provision

**DOI:** 10.1007/s10995-024-03932-4

**Published:** 2024-05-31

**Authors:** Tamarind Russell-Webster, Anna Davies, Miriam Toolan, Mary Lynch, Rachel Plachcinski, Michael Larkin, Abigail Fraser, Sonia Barnfield, Margaret Smith, Christy Burden, Abi Merriel

**Affiliations:** 1 Academic Women’s Health Unit, Bristol Medical School, Second Floor, Learning and Research Building, North Bristol NHS Trust, Westbury-on-Trym, BS10 5NB UK; 2 Centre for Academic Child Health, University of Bristol, 1-5 Whiteladies Road, Bristol, BS8 1NU UK; 3 Academic Women’s Health Unit, Level 2, Learning and Research Building, North Bristol NHS Trust, Bristol, UK; 4 North Bristol NHS trust, Bristol, BS10 5NB UK; 5 NCT, 30 Euston Square, London, UK; 6 Department of Psychology, Aston University, Birmingham, B4 7ET UK; 7 Population Health Sciences, Bristol Medical School, University of Bristol, Oakfield House, Bristol, BS8 2BN UK; 8 Department of Women’s and Children’s Health, North Bristol NHS Trust, Bristol, BS10 5NB UK; 9 Academic Women’s Health Unit, Translational Health Sciences, Bristol Medical School, University of Bristol, Level 2, Learning and Research Building, North Bristol NHS Trust, Bristol, BS10 5NB UK; 10 Centre for Women’s Health Research, Institute of Life Course and Medical Sciences, Faculty of Health & Life Sciences, University of Liverpool, Liverpool Women’s Hospital, Crown Street, Liverpool, L8 7SS UK

**Keywords:** Antenatal care, Maternity services, Health services research, Antenatal education

## Abstract

**Objectives:**

Antenatal education (ANE) is part of National Health Service (NHS) care and is recommended by The National Institute for Health and Care Excellence (NICE) to increase birth preparedness and help pregnant women/birthing people develop coping strategies for labour and birth. We aimed to understand antenatal educator views about how current ANE supports preparedness for childbirth, including coping strategy development with the aim of identifying targets for improvement.

**Methods:**

A United Kingdom wide, cross-sectional online survey was conducted between October 2019 and May 2020. Antenatal educators including NHS midwives and private providers were purposively sampled. Counts and percentages were calculated for closed responses and thematic analysis used for open text responses.

**Results:**

Ninety-nine participants responded, 62% of these did not believe that ANE prepared women for labour and birth. They identified practical barriers to accessing ANE, particularly for marginalised groups, including financial and language barriers. Educators believe class content is medically focused, and teaching is of variable quality with some midwives being ill-prepared to deliver antenatal education. 55% of antenatal educators believe the opportunity to develop coping strategies varies between location and educators and only those women who can pay for non-NHS classes are able to access all the coping strategies that can support them with labour and birth.

**Conclusions for Practice:**

Antenatal educators believe current NHS ANE does not adequately prepare women for labour and birth, leading to disparities in birth preparedness for those who cannot access non-NHS classes. To reduce this healthcare inequality, NHS classes need to be standardised, with training for midwives in delivering ANE enhanced.

## Significance

**What is Already Known on this Subject?:**

There is no detailed national guidance about the content or quantity of antenatal education that should be delivered by the NHS to patients and, how coping strategies are developed within these sessions is unknown.

**What this Study Adds?:**

Antenatal educators believe that quality of antenatal education needs to be improved. Access to coping strategies for labour and birth is hindered by the type of class, the educator’s approach and socioeconomic status. This study can be used to support an argument for addressing the inequality in access to antenatal preparation, whilst better preparing all women through higher quality of antenatal education provision in the NHS.

## Introduction

Antenatal education (ANE) is mandated by the National Institute for Health and Social Care Excellence (NICE) and is seen as an important part of care to prepare people for childbirth and the immediate postnatal period. It is currently offered to women/birthing people (referred to as women henceforth) and their partners by the National Health Service (NHS) (Gokce Isbir et al., 2016a, 2016b; Guidance, 2008; Kacperczyk-Bartnik et al., 2019). In the United Kingdom (UK), whilst the content is not well defined NICE guidelines recommend a focus on childbirth, breastfeeding and immediate postnatal support. Antenatal education is one way in which women and their birth partners can be prepared for birth (Gokce Isbir et al., 2016a, 2016b; Kacperczyk-Bartnik et al., 2019). The “Ready for Child program” found that women who attended antenatal classes reported a more positive birth experience (Maimburg et al., 2010). This is particularly important as one in three women experience some form of birth trauma (Alcorn et al., 2010), factors which may contribute to this include postpartum haemorrhage, emergency caesarean and admission to the neonatal intensive care unit. Good quality birth preparation may be key in managing mental health risks of traumatic delivery, by preparing women for different eventualities of childbirth and enabling them to develop coping strategies to manage pain and deal with changes during the course of their delivery (Alcorn et al., 2010). Traumatic childbirth experiences have been associated with postpartum mental health problems, including depression and post-traumatic stress disorder (PTSD) (Ayers & Pickering, 2001; Taheri et al., 2018). Poor mental health during the postnatal period has significant consequences including attachment disorders and a reduction in breastfeeding rates (Beck & Watson, 2008; Nilsson et al., 2012).

Antenatal education aims to increase birth preparedness through a two-pronged approach. Firstly, it improves overall understanding of childbirth and the likelihood of requiring intervention (Shub et al., 2012). Secondly, it aims to equip individuals with coping strategies, both pharmacological (i.e. pain relief) and non-pharmacological, to cope with pain and emotional distress, during birth and labour (Green et al., 1990). There is a wide variety of non-pharmacological coping strategies that can be used by women to promote coping with childbirth including but not limited to TENS, aromatherapy, acupuncture, massage, hypnosis, labour support, reflexology and labour positions. Supporting women to use these in labour is recommended as part of care within the NICE guidelines and trials evidence their use (Kimber et al., 2008; Liu et al., 2010). However, there is a lack of high-quality research about both which specific coping strategies women find most effective in labour and what opportunities women have to learn and develop these skills (Beverley Griggs, 2019; Brixval et al., 2016; Levett et al., 2016; Prasertcharoensuk & Thinkhamrop, 2004). Approximately 65% of women in the UK are offered NHS antenatal education classes and 23% of primiparous women attend non-NHS classes (Beverley Griggs, 2019). There is currently no available guidance about the content or quantity of antenatal education that should be delivered by the NHS to patients or on how classes should be delivered (Guidance, 2008). Detailed information about the content of current antenatal education provisions, and variations between providers is not available (Svensson et al., 2006).

Antenatal education is delivered by a variety of educators. In the NHS it is often delivered by community midwives and physiotherapists. However, in the private sector there are a greater variety of antenatal educators; some are clinically trained, some are trained by private organisations [e.g. National Childbirth Trust (NCT)] and others provide specific education around skills (e.g. yoga, hypnobirthing). What many of these educators have in common is that they arrange, deliver and advocate for antenatal education. Collectively this group of people has a wealth of experience and an excellent overview of antenatal education provisions in the UK.

This study aims to understand the extent to which educators perceive that current antenatal education supports women to be prepared for childbirth and to identify how they believe the quality of this can be improved to support women in developing coping strategies.

## Methods

A cross-sectional, UK wide, online survey to describe antenatal educators’ perspectives regarding current antenatal care, conducted between October 2019 and May 2020*.* Each topic covered had both multiple choice and open-ended questions.

We asked questions about:

- Respondent demographic characteristics and job role
- Antenatal educator perspectives of adequacy of current antenatal education provisions and the impact of antenatal education on birth preparedness.
- Feedback that antenatal educators have received from women about current NHS antenatal education classes.
- Accessibility of teaching on coping strategies and antenatal educator views about these.
- Antenatal educators’ views on ideal provisions for structure and content for antenatal education classes.

Survey data was collected and managed using REDCap electronic data capture tools hosted at the University of Bristol (Harris et al., 2009, 2019). Table 1 shows the topics discussed and themes elicited from these.

Antenatal educators were purposively sampled. For purposes of anonymity, the organisation of respondents was not recorded however, organisations targeted included: NCT educators, hypnobirthing practitioners and NHS midwives. Antenatal educators were identified via an internet search to discover groups of interest and individuals who are influential, for example heads of community midwifery services, and had to be currently delivering antenatal education either within the NHS or through private practice. Organisations were screened to ensure they were operating in the UK, and were contacted by email with a link to the survey. Antenatal educators were given the option to complete the survey online or through telephone interview, however the majority of information collected was via online survey. Sampling was conducted for diversity across antenatal educational educator groups in terms of type of deliverer or type of ANE delivered.

Respondents’ data were used in the analysis if they had completed at least one survey item other than their demographic characteristics.

Data from the multiple-choice questions were subjected to quantitative analysis. Percentages were calculated using the count as the numerator and the total number of participants who answered that question as the denominator, such that the denominator varied according to question.

Qualitative data derived from the open-ended questions were exported into NVivo for analysis (International 2017). Thematic data analysis involved an iterative process of reading and re-reading questionnaire responses whilst open coding for words and phrases, followed by assigning words and phrases into clusters and then further assigning them to super-ordinate themes (Attride-Stirling, 2001). As a validity check, two further authors independently read questionnaire responses and identified no additional themes. The themes mentioned by the greatest number of antenatal educators were prioritised in the analysis.

Patient representatives were involved in the study steering committee and inputted into the initial study design.

## Results

Survey invitations were sent to 478 antenatal educators; both individuals and organisations. Ninety-nine participants responded, a response rate of 20.7%, with 94 complete and five partially complete responses. There was representation of educators across England, Scotland and Wales. Twenty-five of the survey respondents were qualified midwives and 21 were independent antenatal practitioners.

### Adequacy of Current Antenatal Education Provision to Prepare Women for Labour and Birth

62% of antenatal educators stated that they did not believe NHS antenatal classes prepared women for labour and birth. In the free-text responses to this question the most commonly mentioned themes elicited were: barriers to accessibility of classes; limited class content; and lack of information tailored to individuals.

#### Barriers to Attending Classes

Many antenatal educators described barriers to accessibility of classes as a reason why NHS antenatal education classes do not adequately prepare women for labour and birth. Barriers to accessibility included lack of availability of classes, midweek and daytime classes not being accessible for women with childcare or work constraints, only primiparous women being invited to attend, and language barriers whereby classes are only offered in English. Specific examples from antenatal educators included

“women are not advised to sign up for the antenatal course until they are 30 weeks pregnant, but by this time the antenatal course is already full” [antenatal educator].

One midwife commented that “having worked in the NHS I do not believe there is the time, nor the resources to adequately prepare women, especially when there are language barriers”.

#### Limited Class Content

Many antenatal educators described NHS antenatal education classes as having limited breadth and depth of information with significant emphasis on medical intervention. One private antenatal education teacher described NHS classes as being “biased towards a medical model of birth.” Time constraints were cited as a contributing factor to limited class content. An NHS midwife commented that “the duration of classes presents a barrier for meaningful discussion.”

#### Lack of Individualised Information

Many antenatal educators believed there was a lack of individualised, parent-centred care in antenatal education classes meaning that parents were not aware of all of their options in order to make an informed decision: “Very few women realise the choices they have around the care they are given before, during, or after birth.” Additionally, one antenatal educator described NHS antenatal education classes as a “tick-box exercise” whereby information given is standardised and another educator commented “more individualised person-centred support is needed”.

### Feedback Received from Women About Current NHS Antenatal Education Classes

Seventy-nine antenatal educators (80%) had heard feedback from women about their experiences of NHS antenatal classes. 24% of these commented that women had fed back they didn’t feel adequately prepared for birth. Commonly mentioned themes in feedback received were: the teaching style and quality; and class resources including timings and class size.

#### Teaching Style and Quality

Several antenatal educators said that women felt classes had inadequately prepared them for birth. Many antenatal educators had received feedback about the teaching style in classes. Women felt they would prefer a more interactive and engaging teaching style as opposed to a lecture-based class and that classes needed to be smaller to help with engagement. One private antenatal educator received feedback that “the style was lecture-like rather than exploring what people thought—but this is understandable given the lack of time available.” Additionally, women felt they had few practical techniques to help with birthing: “Women usually say there is a lot of information given and classes do not provide practical techniques.”

Additional feedback alluded to the variability in the quality of teaching between midwives. For example, one piece of feedback commented: “they (midwives) often receive little to no training in teaching or facilitating adult learning. I hear often from parents that the delivery of the information could be greatly improved.” However, other feedback suggested that women valued being taught by midwives: “it was good to hear from the midwives’ experience and opinion.”

#### Class Resources Including Timings and Class Size

Several antenatal educators received feedback that classes had too many people and were too short. One antenatal educator commented that due to the “lack of time available” it was difficult to facilitate discussions and individualise teaching. Another piece of feedback a midwife received was “the sessions were rushed and crowded. That the experience was very much like school, you sat and listened” and another commented “most (women) say the classes were too big, they were too short to get any detailed information and only covered normal birth. [as opposed to medical interventions such as instrumental delivery or caesarean sections]”.

### Teaching Coping Strategies to Support Labour and Birth

A total of 94 antenatal educators responded to the questions addressing the use of antenatal education to equip women with coping strategies for labour and birth. 55% of these antenatal educators believed the opportunity for women to learn about coping strategies for labour and birth varied between location and educators. 35% of antenatal educators believed women did not have adequate opportunity to develop these skills. In the free text response to this question barriers to developing coping strategies were acknowledged with themes relating to: affordability of private provisions; and time constraints.

#### Affordability of Private Provisions as a Barrier to Developing Coping Strategies

The most frequently mentioned barrier to developing coping strategies was affordability of private classes which was mentioned by 32% of stakeholders. Many antenatal educators commented that private birth preparation classes addressed a greater variety of coping strategies than NHS classes but were not always affordable. For example, some antenatal educators, both private and NHS, felt that little time was spent on developing non-pharmacological coping strategies in NHS classes. One hypnobirthing instructor commented that “where women can afford private classes, they have the opportunity to develop coping strategies. Where they cannot afford private classes (the majority of women) they are unable to get the information that they need.”

#### Time Constraints as a Barrier to Developing Coping Strategies

Time constraints were cited as another barrier to developing strategies with one private midwife saying “I don’t think there is enough time in NHS classes to do this, as there is so much to cover in such a short timeframe.”

#### How Best to Support Women to Develop Coping Strategies

Antenatal educators were asked what they thought was the best method to support women to develop coping strategies in the antenatal period. Themes discussed were increased practice of coping strategies throughout the antenatal period; and the roles of health care providers in enabling parents to develop these strategies. Antenatal educators suggested approaches to facilitate increased practice of coping strategies included introducing them earlier in pregnancy and increasing the frequency of practicing coping strategies both in a home environment and healthcare setting. Antenatal educators also mentioned that the actions of healthcare providers played an important part in preparing women to use coping strategies during labour and birth for example, the non-biased presentation of available coping strategies regardless of the attitudes of educators towards the coping strategy. One private midwife said “women have to be given all of the tools in the toolbox and then they have to learn which ones are their favourite and become familiar with them. They need to know and be educated about them all.”

### Supporting the Utilisation of Coping Strategies

#### How Healthcare Professionals Can Support Women to Use Coping Strategies During Labour and Birth

Antenatal educators described the need for: a parent-centred, individualised approach; Continuity of Care (COC) and knowledge of healthcare professionals about a wide range of coping strategies. A parent-centred approach, whereby healthcare professionals are aware of a woman’s birth plan and preferences was the most commonly mentioned theme. This encompasses where women want to labour, whether they want to have a vaginal delivery or caesarean section and their analgesia preferences. COC was cited as a useful strategy to help with parent-centred care with one hypnobirthing teacher suggesting COC is important so that “professionals can understand a woman’s preferences”. Many antenatal educators felt that the healthcare professional having a wide range of knowledge surrounding non-pharmacological and pharmacological coping strategies is also important.

#### The Most Useful Strategies to Support Coping in Labour

**Fig. 1:**
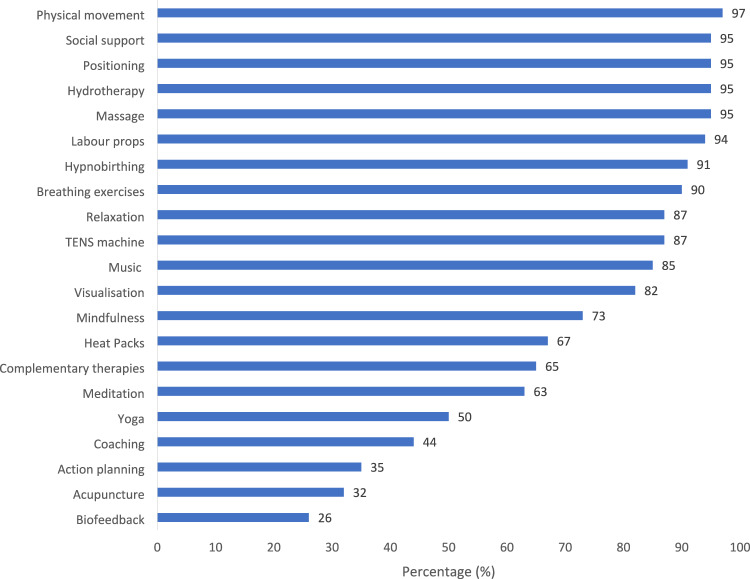
Antenatal educator opinions of what coping strategies women find useful during labour, n = 94

When asked which coping strategies antenatal educators thought women find most useful, physical movement was perceived as the most helpful and yoga least helpful (Fig. 1). We provided the opportunity to suggest additional coping strategies. Antenatal educators suggested: self-care strategies (rest, nutrition, and hydration), aromatherapy and distraction therapies.

#### Birth Partners and Coping Strategies

Forty-seven antenatal educators (50%) believed that birth partners do not have the opportunity to learn about coping strategies to support women and 59 antenatal educators (63%) believed that there is not opportunity for birth partners to develop coping mechanisms to support themselves.

### Structure and Content of Antenatal Education Classes

**Fig. 2:**
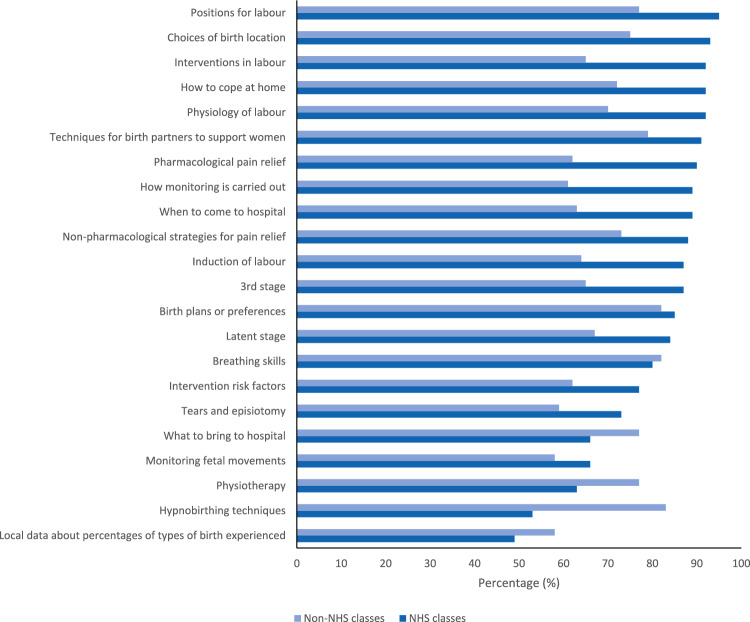
Antenatal educators’ opinions on what topics should be included in both NHS and non-NHS classes, n = 99 United Kingdom 2020

**Fig. 3:**
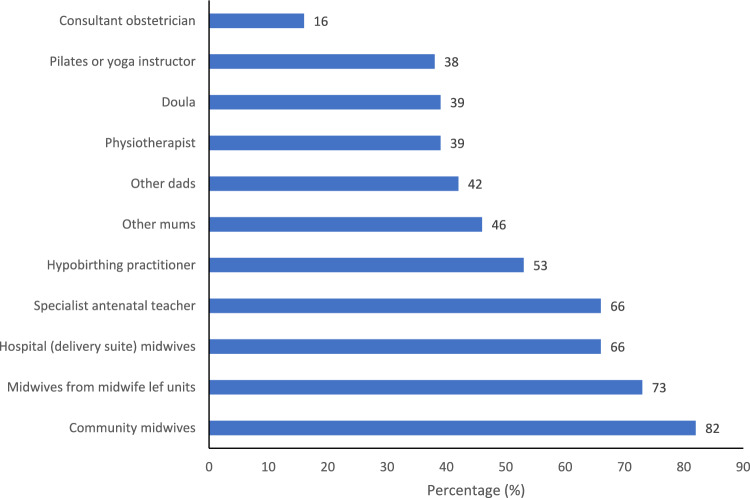
Antenatal educators’ opinions of who women should hear from during NHS antenatal education classes, n = 99, United Kingdom 2020

Antenatal educators were asked how many hours of antenatal education are needed and realistic within the NHS budget to enable birth preparation. 19% of antenatal educators thought that up to 4 h of education was required, 33% thought that up to 5 h of education was needed and 29% suggested that more than 5 h are required. With regards to topics that are essential to be included in NHS or private classes there was variation in what antenatal educators’ thought were priority topics. Positions for labour and choice of birth location were seen as the most important topics to cover in NHS classes. Meanwhile hypnobirthing techniques, breathing techniques and focus on awareness of choice with regards to birth plans or preferences were seen to be the most important topics to include in private classes (Fig. 2). Antenatal educators suggested that the most appropriate healthcare professional for women to hear from during classes was community midwives. Antenatal educators thought the least appropriate healthcare professional for women to hear from was consultant obstetricians (Fig. 3).

## Discussion

Antenatal educators have highlighted that the current NHS antenatal education provision is inadequate for preparing women for labour and birth. They described practical barriers (e.g. timing, availability, language barriers) and barriers to quality (e.g. limited time, lack of individualised, parent-centred care, poor delivery/teaching preparation). Their assessment is that coping strategies are not taught widely throughout NHS antenatal classes but are often taught by private providers, which creates inequality in provision. Despite these concerns antenatal educators believed that different topics should be covered in NHS and private classes.

Our study found practical barriers to the accessibility of NHS antenatal classes. Greater focus on planning the classes to suit the needs of individuals and their partners is needed to improve this. This may include class times outside of the working day, online classes and widening access to multiparous women for whom classes are sometimes not available and making classes accessible for people who do not speak English. In addition, antenatal educators believed that there are inequalities in access to antenatal education provision (both NHS and non-NHS). This was also seen in a antenatal education care review which found that women in the most and least deprived quintiles and Black, Asian and Minority Ethnic (BAME) communities were less likely than other women to have been invited to attend classes (Beverley Griggs, 2019). Morton and Simkin report “health maintenance for all not just for the richest” is an important part of respectful maternity care (Morton & Simkin, 2019). This is particularly emphasised by the fact that women who are least likely to access private classes (young people, BAME groups, lower socioeconomic classes) are also the people who are at greatest risk of morbidity and mortality (Beverley Griggs, 2019), and so are perhaps the people whom the NHS needs to target the most when considering antenatal preparation for birth. One suggestion could be the training of educators from the same communities as women who are least likely to attend, so that antenatal education can be delivered in the native language of people attending the class and would also mean that the educator could have a better knowledge and cultural understanding of the challenges that may impact these specific groups.

In addition to the discrepancy in the quality of information delivered between NHS and private providers, and the lack of adequate opportunity to develop coping strategies, antenatal educators were also concerned that NHS ANE covers a narrower range of topics and is more medically focussed. This is likely secondary to the barrier of lack of time, for example, for a woman to be taught hypnobirthing and to have the opportunity to practice it requires more time. Access to a narrower range of topics may lead to a woman, especially one who can only afford to access NHS classes, being less aware of options during labour and make her feel less involved in the decision-making process. A lack of involvement in decision making during labour has been shown to contribute to negative birth experiences including poorer postnatal mental health outcomes (Elmir et al., 2010; Olde et al., 2006; Thomson & Downe, 2010). A standardised curriculum may improve the accessibility of a wider range of topics in a more time efficient way. While it is acknowledged that there is not currently funded time to deliver additional content, it may be possible to reduce this inequality by within the standardised curriculum highlighting basic resources to women.

We found that antenatal educators believe NHS classes sizes to be too large, creating a barrier for meaningful discussion. Other research has also shown that large class groups create an obstacle to a participatory educational approach (O’Sullivan et al., 2014). To improve how classes are planned and structured, further research should involve speaking to pregnant and recently post-partum individuals who have recent lived experiences of attending antenatal classes to find out their specific needs and allow them to voice suggestions for the shape of educational opportunities within their journey. As such, a standardised curriculum can be devised which prioritises the content which pregnant women find most important in a time efficient way that is useful for the patient.

Results from our study suggest that there is variation in class facilitation skills between educators which may further drive inequalities in the antenatal education provision across the UK. This was similarly found in an observational study by Cutajar and Cyna which found variability in the content and time taken for information delivery in antenatal classes between midwives (Cutajar & Cyna, 2018). Within the public sector, there is no specific training in this area and community midwives may be required to deliver education as part of their job role, whether they are accomplished class facilitators or not. This may be improved through having specific midwives to be trained to deliver education rather than it being the role of every midwife by default and additionally, educators should be delivering education on topics about which they are confident for example, labour ward midwives or consultant obstetricians may be better suited to deliver education around medical interventions such as the use of forceps than community midwives.

### Strengths and Limitations

To the authors’ knowledge, no other studies have examined perspectives of antenatal educators of antenatal education in the UK. Strengths of our study included a mixed methods approach which allowed us to explore further the reasons for participants’ responses. Additionally, we received responses from both NHS and non-NHS antenatal educators who deliver a broad variety of antenatal classes, with good representation from across England, Scotland and Wales. A wide range of professionals are involved in the delivery of ANE, we believed it was important that all of their voices are heard. The paper also covers what is included in ANE, it does not however, consider the practicalities of how the hospital is prepared to support the woman in her choices during labour and birth. We acknowledge that the support from different professionals may be varied however, within the NHS there is much support from the midwifery community for using a wide range of coping strategies (Merriel et al., 2023). Additionally, there are other topics which may be important to cover in ANE that are not currently included in the broad guidelines for antenatal education provided by NICE. This could include respectful maternity care and addressing issues such as intimate partner violence, further research to design a standardised curriculum may consider including these topics.

The response rate of 21% to our study was not high. However, this is in keeping with return rates with other online surveys, and even with a higher response rate, we feel there would have remained an element of self-selection whereby our research is more likely to be impacted by those who are more motivated to be involved with childbirth education (Hendra & Hill, 2019; Iversen et al., 2020; Khazaal et al., 2014). The study only takes in the views of antenatal educators, not people attending classes. We accessed both NHS and private providers because we believed it was important to hear about provision in both sectors.

## Conclusion

Antenatal educators believe current antenatal education provision in the UK does not adequately prepare women for labour and birth. There is an inequality in the level of information, quality of delivery and accessibility of ANE between NHS and private providers. To reduce this healthcare inequality, a standardised minimum curriculum for NHS classes is needed and training on delivery of this education for midwives needs to be enhanced so that high-quality education is available to everyone.

Future research should investigate the views of pregnant women and their partners about current antenatal education provisions in the UK to establish their needs and define a high-quality, evidence-based NHS curriculum.

## Data Availability

Data available on request with appropriate ethical approvals.

## Appendix

See Table 1, Figs. 1, 2, 3.

**Table 1:** The topics discussed, and the main themes elicited from these

Topics discussed	Main themes elicited
Adequacy of current antenatal provisions	• Barriers to attendance • Limited content • Lack of parent-centred care
NHS class feedback	• Teaching style and quality • Class resources
Teaching about coping strategies	• Affordability of private provisions • Time constraints
Supporting utilisation of coping strategies	
